# Evaluation of a digital health platform for preventing stroke in the Australian community: Study protocol for a randomized controlled trial – Love Your Brain

**DOI:** 10.1371/journal.pone.0330868

**Published:** 2025-09-04

**Authors:** Monique F. Kilkenny, Dominique A. Cadilhac, Amanda G. Thrift, Mark R. Nelson, Janet Bray, Jan Cameron, Timothy Kleinig, Muideen T. Olaiya, Lisa Murphy, Tara Purvis, Rosanne Freak-Poli, Catherine Burns, Christine Farmer, Belinda Bullas, Lachlan L. Dalli, Eleanor Horton, Brenda Booth, Stephanie Ho, Seana L. Gall

**Affiliations:** 1 Stroke and Ageing Research, Department of Medicine, School of Clinical Sciences at Monash Health, Monash University, Clayton, Victoria, Australia; 2 Stroke and Critical Care Research Theme, Florey Institute of Neuroscience and Mental Health, University of Melbourne, Heidelberg, Victoria, Australia; 3 Menzies Institute for Medical Research, University of Tasmania, Hobart, Tasmania, Australia; 4 School of Public Health and Preventive Medicine, Monash University, Melbourne, Victoria, Australia; 5 Department of Neurology, Royal Adelaide Hospital, Adelaide, South Australia, Australia; 6 Department of Medicine, University of Adelaide, Adelaide, South Australia, Australia; 7 Stroke Foundation, Melbourne, Victoria, Australia; PLOS: Public Library of Science, UNITED KINGDOM OF GREAT BRITAIN AND NORTHERN IRELAND

## Abstract

**Rationale:**

One in four people will have a stroke in their lifetime. Over 80% of strokes are preventable through the management of modifiable risk factors. There is a growing demand from the community for information about how to prevent stroke. The Love Your Brain digital platform comprises an online course (Massive Online Open Course) and text messages to improve stroke knowledge and motivate behaviour change for stroke prevention.

**Aims:**

To determine the effect of the digital platform vs a control on attendance at a medical practitioner for cardiovascular risk assessment or management, from either a general practitioner or specialist.

**Methods and design:**

Love Your Brain is a Phase III, prospective, single-blinded three-arm randomized controlled trial. Eligible participants are community-dwelling residents of Australia aged ≥45 years, who communicate in English language, can access internet and a smartphone, and do not have a self-reported history of stroke or major cardiovascular event. Participants are randomised to either receive the online course, text messages, or general information about stroke risk factors via email (control). Online surveys will be conducted at baseline and 12 weeks. Outcomes will be assessed based on intention-to-treat analysis. Self-reported medical visits will be validated using data linkage. Process and economic evaluations will be conducted in parallel to the trial. An independent statistician blinded to group will analyse the data.

**Study outcomes and sample size:**

The primary outcome is a visit to a medical practitioner for cardiovascular risk assessment or management within 12-weeks of randomization. Secondary outcomes include: (1) knowledge of stroke signs and risk factors; (2) healthy or risk-modifying behaviours; (3) adherence to medications; (4) process evaluation including intervention delivery/implementation and satisfaction; (5) economic evaluation including health care resource use and cost; and (6) adverse events. Assuming 80% power (two-sided α = 0.05) and 40% prevalence in the control group, 894 participants (298 for each of three groups) will be required to detect a 30% relative increase in medical practitioner attendance for cardiovascular risk assessment or management from either a general practitioner or specialist in the intervention groups.

**Discussion:**

This study will provide evidence for the efficacy of a low-cost digital health intervention (online course or text messages) to reduce the risk of stroke in the community.

**Trial registration:**

ACTRN12625000124437

## Introduction

Stroke is common, affecting an estimated 1 in 4 people in their lifetime [1]. In 2023, over 45,000 strokes occurred in Australia with a cost of $9 billion to the economy [2]. At least 80% of strokes are preventable through effective management of risk factors such as high blood pressure, smoking, high cholesterol, inadequate diet and physical inactivity [1,3]. Stroke prevention has been prioritized in Australia [4] due to the high prevalence of cardiovascular risk factors [5] and inadequate reach and efficacy of existing primary prevention strategies [6–8].

There is growing evidence and acceptability of the use of digital health interventions for primary stroke prevention [9–12], but limitations include poor adherence, and lack of stroke awareness [13].

The Love Your Brain digital platform was designed to provide evidence-based information to help the community ‘take action’ to identify and manage their risk factors for stroke. The digital platform comprises an online course (Massive Open Online Course) and health education text messages. The content and delivery of the digital platform was co-designed with health knowledge experts and community members [14,15] and adapted from previous work in secondary stroke prevention [16]. The digital platform incorporates evidence-based information and personalized content to motivate behaviour change, aligned with international stroke prevention guidelines [17].

A feasibility pilot study (n = 31 participants) was conducted to test recruitment methods, enrolment processes, user satisfaction and content acceptability of the digital platform. Results of mixed methods process evaluation indicated that participants were generally positive about their involvement, most (94%) would recommend the digital platform (online course or text messages) to others and most (79%) agreed that a digital platform to learn about prevention of stroke is a good idea. Participants valued the flexibility of the online course and convenience of the text messages, and a 77% retention rate was achieved.

Our primary aim is to determine the efficacy of the Love Your Brain digital platform in improving attendance at a medical practitioner for cardiovascular risk assessment or management, from either a general practitioner or specialist, compared to a control at 12-weeks post-randomization.

## Materials and methods

### Design

**Fig 1:**
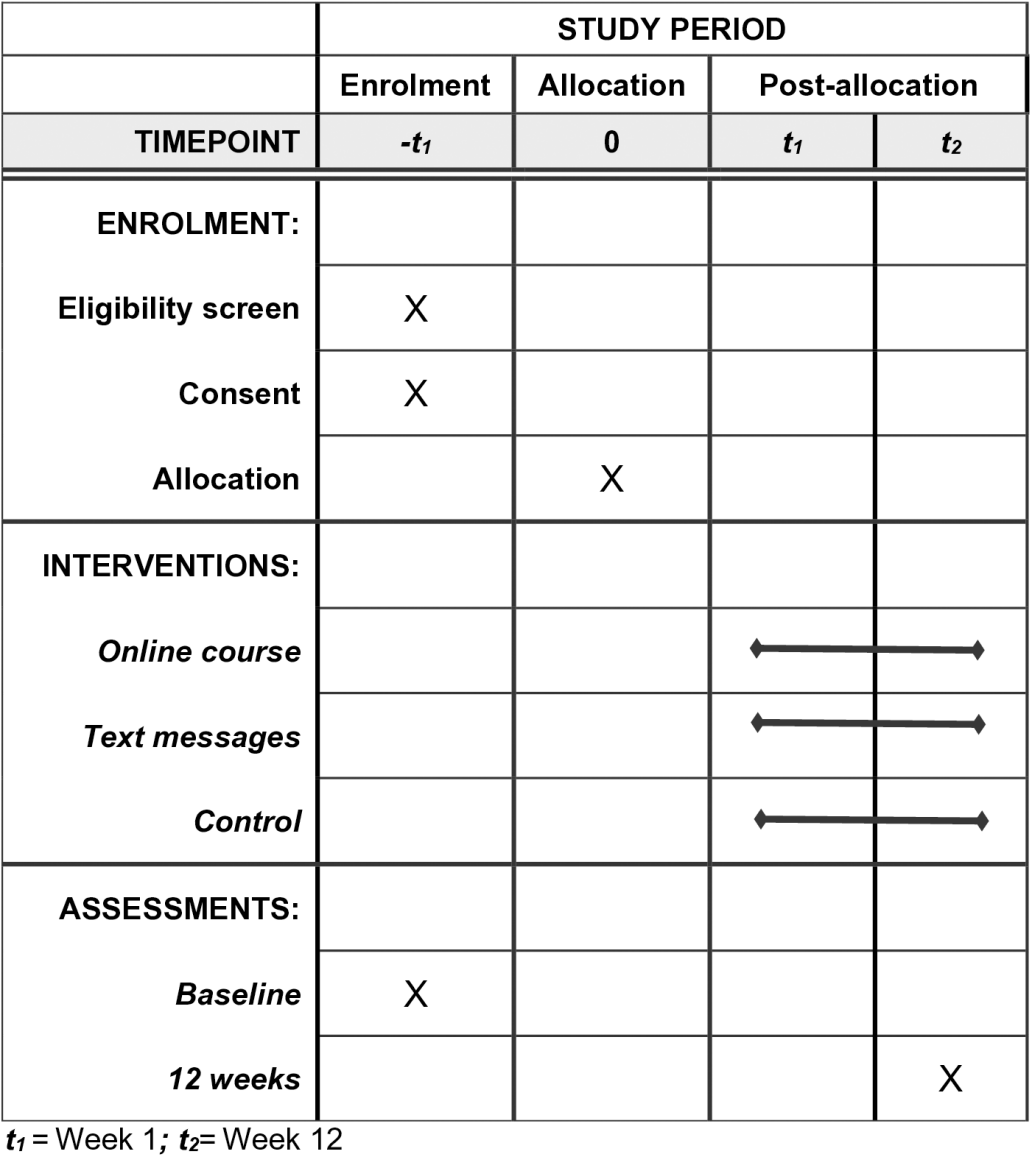
SPIRIT schedule for Love Your Brain: enrolment, interventions, and assessments.

Love Your Brain is a Phase III, prospective, single-blinded, three-arm randomized controlled trial being undertaken in Australia. This protocol aligns with the Standard Protocol Items: Recommendations for Interventional Trials (SPIRIT) [18]. The completed SPIRIT schedule is shown in Fig 1.

### Participant eligibility

Participants are recruited through advertising and Stroke Foundation StrokeSafe education presentations [19]. Participants in the pilot study indicated they wanted more information about the digital platform before enrolment. Therefore, participants will be unblinded to the intervention groups, however, they will be informed that allocations to a specific delivery modality (online course, text messages or emails [control]) is via randomization and cannot be chosen.

Inclusion criteria:

- Aged 45 years or more
- Able to communicate in the English language
- Residing in Australia
- Able to access the internet and a smartphone
- Have watched a StrokeSafe presentation (in-person or online) in the last 12 weeks

Exclusion criteria:

- History of stroke or major cardiovascular event (self-reported; includes heart attack/myocardial infarction, coronary artery bypass surgery)

Screening is conducted online using REDCap, a secure web-based data management system [20,21]. Participants who consent to participate in the trial are asked to complete baseline assessment questionnaires for demographic, health and lifestyle factors (Table 1).

**Table 1:** Study data collection timeline including outcomes.

Data collection (self-reported)	Baseline	12 weeks
Demographic and socioeconomic information	X	
Cardiovascular risk factors	X	X
Selection of healthy choices to manage risk factors	X	
*Primary outcome*		
Medical practitioner attendance for cardiovascular risk assessment or management, from general practitioner or specialist	X	X
*Secondary outcomes*		
Stroke knowledge (Stroke Knowledge Test [24])	X	X
Identification of health and lifestyle risk factors	X	X
Physical activity (IPAQ [25])	X	X
Diet (Mini-Eats [26])	X	X
Body-mass index	X	X
Smoking cessation	X	X
Alcohol consumption	X	X
Sleep and wellbeing (EQ-5D-5L-Psychosocial [27,28])	X	X
Medication adherence (MARS-5 [29])	X	X
*Process and economic evaluation*		
Satisfaction and evaluation survey		X
Spend on health behaviours	X	X
Healthcare resource utilisation	X	X
Adverse events		X

IPAQ, International Physical Activity Questionnaire; MARS-5, Medication Adherence Report Scale-5.

### Randomization

Following the baseline assessment, participants are automatically randomized to one of three intervention groups in 1:1:1 ratio (online course intervention: text message intervention: control group). Randomization is undertaken through the REDCap with stratification balancing by age (45–64 years, ≥ 65 years) and gender (man, woman, non-binary/gender diverse/prefer not to say).

### Intervention

**Fig 2:**
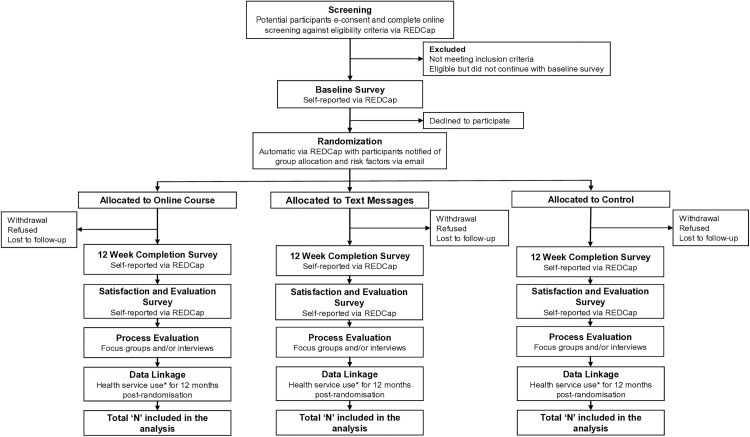
Conceptual model for the Love Your Brain digital platform. *For participants who have consented to the release of Medicare Benefits Schedule and Pharmaceutical Benefits Scheme data.

There are two intervention groups in this trial (Fig 2). All three groups receive three administrative messages, including a welcome message with a summary of their self-reported risk factors, 12-week completion survey invitation, and a thank you message at the conclusion of their participation. The intervention groups receive content to improve knowledge of stroke signs and risk factors, and encourage health behaviour change to prevent stroke.

One intervention group receives an ***online education course*** (Massive Online Open Course) delivered over eight weeks. Modules are self-paced and participants are expected to complete seven core modules and two elective modules. Core modules include (1) Introduction; (2) What is stroke?; (3) Stroke numbers; (4) Signs of stroke – F.A.S.T; (5) Risk factors for stroke; (6) Action plan; and (7) Completion module. Each participant will then elect (or be guided based on self-reported risk factors and interests) to complete at least one module based on biomedical risk factors for stroke (specifically blood pressure, cholesterol, atrial fibrillation, overweight and obesity, blood sugar and diabetes); and at least one module based on lifestyle-based behavioural risk factors for stroke (specifically smoking, diet and alcohol, exercise, sleep and wellbeing). Modules include short videos with transcripts, text, links to further information and knowledge quizzes. Module duration ranges between 30 minutes to 1 hour, with the complete course being achievable in 4 hours.

The second intervention group will receive ***health education text messages*** that are personalized to the participant’s risk factors of interest as identified in the baseline survey. Participants will receive between 31 and 61 messages over 12 weeks, delivered by SMS or email according to their preference. Participants can choose to receive messages for between one and four risk factors, with the number of risk factors influencing the total number of messages received. The text messages are in plain language with the vast majority (98%) achieving a readability index of Grade 10 or less (79% ≤ Grade 8). Links to additional resources are provided in some text messages. Development of the text message intervention is detailed elsewhere [22].

The control group will receive five generic emails fortnightly about stroke risk factors (blood pressure, diet, alcohol, exercise, smoking) with links to relevant Stroke Foundation webpages. These resources are freely available to the general public and therefore align with ‘usual care’.

Participants can withdraw from the study at any time. All participants will be predominately contacted through automated email or SMS reminders from the REDCap database [20,21], and online course systems. Using the Dillman protocol [23], three attempts at contact will be made to improve response rates. Where there is non-response (either survey non-completion or not completing the online course modules), study staff will contact participants by phone and SMS to encourage continued participation. Study staff contacting participants at 12-weeks will be blinded to the group allocation. Participants who complete the 12-week completion survey can enter a prize draw to win one of five $200 supermarket e-vouchers.

### Outcome assessments

Participants self-report primary and secondary outcomes through online surveys via REDCap [20,21] at baseline and after the 12-week intervention period (Table 1).

#### Primary outcome.

The primary outcome is a visit to a medical practitioner for cardiovascular risk assessment or management, from either a general practitioner or specialist, within 12 weeks of randomization. This outcome will be self-reported by participants and subsequently validated using the Medicare Benefits Schedule (MBS) and Pharmaceutical Benefits Scheme (PBS) codes for consenting trial participants.

#### Secondary outcomes.

The secondary outcomes are self-reported knowledge of stroke signs and risk factors, uptake of healthy or risk-modifying behaviours, and adherence to medications (Table 1).

Stroke knowledge is assessed as any change in stroke knowledge measured using the Stroke Knowledge Test [24], incorporating stroke risk, warning signs and appropriate emergency responses to a suspected stroke.

Uptake of healthy or risk-modifying behaviours:

- Physical activity measured by the International Physical Activity Questionnaire [25]. Physically active is defined as self-reported ≥30 minutes of moderate-intensity physical activity, or ≥20 minutes of vigorous-intensity physical activity, at least 3 times a week;
- Diet measured by the Mini-EATS [26], with a healthy eating score of >69;
- Body-mass index with not overweight defined as self-reported body-mass index of <25;
- Smoking cessation measured as self-reported non-smoker of tobacco products;
- Alcohol consumption measured as self-reported standard drinks per week. Risky drinking is considered ≥10 standard drinks per week or ≥4 standard drinks per day;
- Sleep and wellbeing measured by the EQ-5D-5L-psychosocial [27,28].

Adherence to medication will be measured using the Medication Adherence Report Scale-5 [29] and validated using PBS data.

#### Process and economic evaluation.

A mixed-methods process evaluation and economic evaluation will be conducted in parallel to the main trial. The process evaluation will utilize satisfaction survey responses and semi-structured interviews/focus groups to explore implementation and participant experiences. Health care resource use and cost will be gathered from data linkage and surveys to inform the economic evaluation.

### Data monitoring

As this is a low-risk trial that does not involve a therapeutic product, and there are no perceived risks to participants, a data safety monitoring committee is not required. Participants will report adverse events at 12 weeks. An independent Medical Monitor will adjudicate serious adverse events that are deemed to be related to the trial. All data will be collected and stored in a secure REDCap database.

### Sample size estimates

The Love Your Brain study will be powered to detect meaningful differences in the primary outcome. Assuming 80% power (two-sided α = 0.05) and a 40% prevalence in the control group, 894 participants (298 per group) will be adequate to detect a ≥ 30% relative increase in medical practitioner attendance for cardiovascular health in the intervention groups [30,31]. As 57% of those screened were recruited to the pilot study, we estimate screening 1,569 people to achieve the sample size.

### Statistical analysis

The two intervention groups will be compared to the control group. A direct comparison between the online course and text message interventions is not intended, as both will be available concurrently in real-world settings. Standard descriptive statistics will be performed. Between-group differences will be determined using chi-square test for categorical variables and appropriate statistics for continuous variables (based on the distribution of data). We will examine the mean (SD) and/or percentage change in the stroke knowledge assessment tool [32], individual health behaviours and medication adherence using paired t-tests or chi-square tests. We will use mixed models to evaluate the efficacy of the intervention to improve medical practitioner attendance for risk factor assessment or management. The effect of the intervention on change in knowledge of risk factors for stroke and the signs of stroke will be evaluated using generalised linear regression models. Statistical significance will be set as a two-sided p-value of ≤0.05.

### Study organization and registration

The study is coordinated by Monash University, in partnership with Menzies Institute for Medical Research (University of Tasmania) and Stroke Foundation. The Management Committee is responsible for oversight of the trial including design, conduct, analysis and publication. This trial is registered with Australian New Zealand Clinical Trials Registry (Universal Trial Number U1111-1305–2964; ACTRN12625000124437) and approved by Monash University (#45883) Human Research Ethics Committee. Digital consent was used in this trial to obtain participants’ agreement to take part in this trial using a click-to-consent button within REDCap after the participant read the consent form and explanatory statement.

### Recruitment period

The current status of the trial is as follows: recruitment commenced on February 18, 2025 and is expected to end by August 2025. Follow-up is ongoing, and trial results are expected in early 2026.

## Results and discussion

The Love Your Brain project is aimed at reducing the prevalence of stroke across Australia by harnessing the power of digital health to help people identify and manage their risk factors for stroke. The trial has been designed to replicate real-world conditions, with participants able to complete the entire trial independently online, without direct contact from research staff. This trial will address a gap in primary prevention of stroke by providing easily accessible and personalized information in a digital format.

The digital platform was designed with the intention that it would be embedded within the existing Stroke Foundation prevention programs, with free access for the public. The scalable nature of the digital platform means that many more people can be reached by Stroke Foundation including those living in rural and remote communities, at lower cost than with the existing StrokeSafe program.

## Conclusions

The Love Your Brain trial will compare a digital platform (online course and text messages) to a control for primary prevention of stroke. This trial will provide evidence for the efficacy of the digital platform for cardiovascular risk factor assessment and management, stroke knowledge, and uptake of healthy behaviours in the community.

## Supporting information

S1 File Love Your Brain Stage 3 Protocol V1.0 18 Dec 2024. (PDF)

S2 File Love Your Brain Stage 3 Protocol V1.1 25 Mar 2025. (PDF)
