# Supplementary material for: Evaluation of a digital health platform for preventing stroke in the Australian community: Study protocol for a randomized controlled trial – Love Your Brain
Source: PLoS One. 2025 Sep 4;20(9):e0330868. doi: 10.1371/journal.pone.0330868 (PMC12410737; doi:10.1371/journal.pone.0330868)
Supplement: S2 File — (PDF) [file pone.0330868.s002.pdf]

LOVE YOUR BRAIN

A DIGITAL HEALTH PLATFORM FOR PREVENTING STROKE

PROTOCOL – STAGE 3

Effectiveness Randomised Controlled Trial with Process Evaluation and Economic Evaluation

Version 1.1

March 2025

Stroke and Ageing Research Group

Monash University

Administering institution:
Monash University
Wellington Road
Clayton 3800 Australia

Acknowledgement of Country
The members of the Love Your Brain project acknowledge the Traditional Owners of Country throughout Australia. We pay our respects to Elders, past and present.

Acknowledgement of people with a lived experience
The members of the Love Your Brain project acknowledge people with a lived experience of stroke, including their carers and support people. We also acknowledge Stroke Foundation Consumer Council members and lived experience contributors who have provided valuable input into the scope, content and structure of this protocol.

PROJECT TEAM

Any queries about this protocol should be directed to the coordinating Principal Investigator:

Prof Monique Kilkenny
Big Data, Epidemiology and Prevention Division
Stroke and Ageing Research
School of Clinical Sciences at Monash Health
Monash University
Phone: +61402449503
Email: Monique.Kilkenny@monash.edu

Name	Institution	Role in project
Chief Investigators		
Prof Monique Kilkenny	Monash University	Project Coordinating Co-Principal Investigator
Prof Seana Gall	Menzies Institute for Medical Research	Project Coordinating Co-Principal Investigator
Prof Dominique Cadilhac	Monash University	Co-investigator, Senior public health researcher
Prof Amanda Thrift	Monash University	Co-investigator, Senior public health researcher
Prof Janet Bray	Monash University	Co-investigator, Senior public health researcher
Dr Jan Cameron	Monash University	Co-investigator, Senior digital health researcher
Dr Muideen Olaiya	Monash University	Co-investigator, Project statistician, Cardiovascular epidemiologist
Prof Tim Kleinig	University of Adelaide	Co-investigator, Neurologist, Researcher
Dr Lisa Murphy	Stroke Foundation	Co-investigator, Chief Executive Officer, Advocacy
Dr Tara Purvis	Monash University	Co-investigator, Process evaluation, Mixed methods researcher
Prof Mark Nelson	Menzies Institute for Medical Research	Co-investigator, General practitioner, Researcher
Consumer Advisors		
Dr Eleanor Horton	Stroke Foundation	Associate investigator, Clinician, Carer with lived experience
Ms Brenda Booth	Australian Stroke Coalition	Associate investigator, Person with lived experience
Ms Steph Ho		Associate investigator, Person with lived experience
Project Team		
Dr Rosanne Freak-Poli	Monash University	Project Manager
Catherine Burns	Monash University	Project Coordinator
Christine Farmer	Menzies Institute for Medical Research	Online Course Coordinator
Dr Lachlan Dalli	Monash University	Early-career researcher, Epidemiologist

Belinda Bullas	Stroke Foundation	StrokeSafe Program coordinator, Stroke Prevention Manager
Dr Joosup Kim	Monash University	Economic evaluation, Health economist
Furley Johnston	Menzies Institute for Medical Research	Participant follow-up, Research Assistant
Samiksha Dhananjay	Monash University	Participant follow-up, Research Assistant
Eric Kuo	Monash University	Trial dashboard, Research Assistant
Aastha Gurung	Menzies Institute for Medical Research	PhD Student
Kiran Bam	Monash University	Participant follow-up, Research Assistant

Funding

This project is funded by a Medical Research Future Fund Cardiovascular Health Mission grant (#2015976) and NHMRC Synergy Grant STOPstroke (#1182071) awarded by the Australian Government.

Statement of Compliance

This project will be conducted in compliance with this protocol, the National Statement on Ethical Conduct in Human Research (2007) and the Australian Code for Responsible Conduct of Research (2007) produced by the National Health and Medical Research Council of Australia, and regulatory requirements of local and national Human Research Ethics Committees.

Version history:

Version	Date:	Summary of changes
1.0	14/01/2025	Original
1.1	25/03/2025	Added Kiran Bam to the Project Team<br>Appendix F: Updated StrokeSafe Speaker Handbook<br>Appendix G: Updated recruitment materials<br>Appendix J: Updated to final consent form as approved by Services Australia

Contents

Project Team ... 3
1. Acronyms and abbreviations ... 7
2. Background ... 7
3. Aims and objectives ... 11
4. Methods ... 13
4.1 Participants ... 13
4.2 Intervention Arms ... 13
4.2.1 Online Course Intervention ... 13
4.2.2 Text Message Intervention ... 14
4.2.3 Minimal Active Control ... 14
4.3 Sample Size, Recruitment, Consent and Randomisation ... 15
4.3.1 Sample size ... 15
4.3.2 Recruitment ... 16
4.3.3 Consent ... 17
4.3.4 Randomisation ... 18
4.3.5 Blinding ... 18
4.3.6 Withdrawal ... 19
4.3.7 Follow-up procedures ... 19
5. Data collection and Process Evaluation ... 20
5.1 Baseline Survey ... 20
5.2 12-Week Completion Survey ... 22
5.3 Data Linkage ... 22
5.4 Program Evaluation ... 22
5.4.3 Economic Evaluation ... 25
6. Data Analysis ... 25
7. Ethical considerations ... 26
7.1 Privacy and confidentiality ... 26
7.2 Participant Safety ... 26
7.2.1 Adverse Events ... 27
7.2.2 Protocol Breaches ... 28
7.2.3 Disclaimer ... 28
7.3 Data Security and Storage ... 29

7.4 Proposed Dissemination of Results 29
8. Project timeline 30
9. References........ 31

1. ACRONYMS AND ABBREVIATIONS

GP	General practitioner
HREC	Human Research Ethics Committee
MBS	Medicare Benefits Schedule
MOOC	Massive Open Online Course
MRFF	Medical Research Future Fund
PBS	Pharmaceutical Benefits Scheme
QR code	Quick response code
RCT	Randomised controlled trial
SMS	Short message service

2. BACKGROUND

Stroke is common, affecting an estimated 1 in 4 people in their lifetime.[1] Fortunately, stroke is also highly preventable. Of the 45,000 strokes that occur in Australia each year at least 36,000 of these are preventable through effective management of risk factors such as smoking, inadequate diet, high blood pressure and physical inactivity.[2,3] In 2023, the direct costs of stroke to the Australian economy was estimated to be $9 billion, with an expected lifetime cost exceeding $15 billion per person with stroke.[2]

Love Your Brain: A stroke prevention digital platform (ID# 2015976) is a three-year research project to develop and test a digital platform to improve the health knowledge of Australians in stroke prevention (**Figure 1**). It is a collaboration between Monash University, the Menzies Institute for Medical Research (Tasmania) and Stroke Foundation. The project is funded by an Australian Government Medical Research Future Fund (MRFF) Cardiovascular Health Mission targeted call in 2021 (3 years, ID# 2015976).

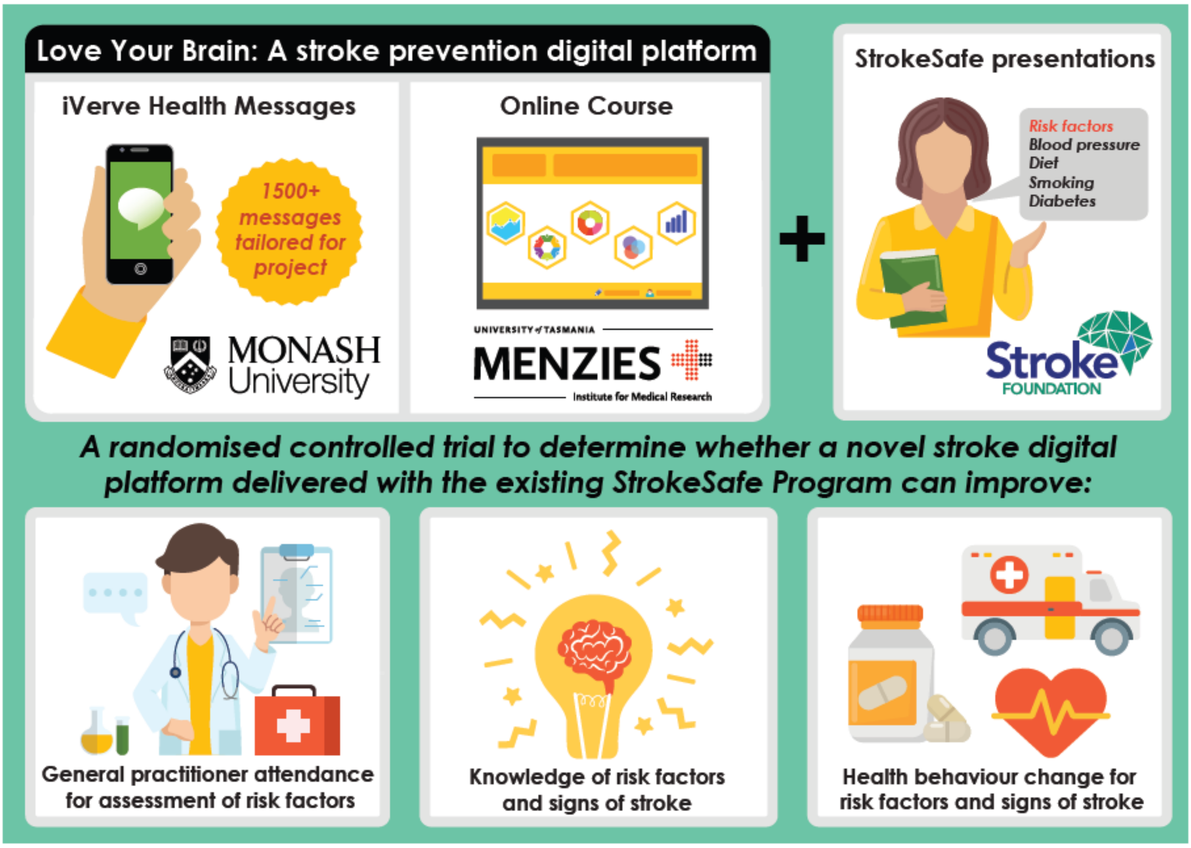

Figure 1: Love Your Brain infographic

We aim to reduce the prevalence of stroke across Australia by harnessing the power of digital health to help people identify and manage their risk factors for stroke. The implementation of our program and subsequent prevention of strokes will be potentially cost-saving. Simply reducing uncontrolled blood pressure alone has been projected to save the Australian health system 315 million dollars a year by decreasing the incidence of stroke.[2]

As described in the project methodology, our digital health platform will go beyond improving the management of one risk factor. Instead, it will equip Australians with the knowledge they need to tackle all risk factors for stroke. Therefore, the economic impacts of our digital health platform are likely to be substantial. The ability to have a large impact is driven by our partnership with Stroke Foundation and their successful StrokeSafe Program (**Appendix A**), which our platform leverages. The StrokeSafe program has been running since 2010 and is undoubtedly successful with over 606 presentations delivered in 2023, reaching more than 21,000 Australians. The StrokeSafe presentations are delivered by volunteers, most of whom have lived experience of stroke. The presentations are delivered in a group setting, and provide education for primary stroke prevention. The StrokeSafe presentations have been effective in improving knowledge of stroke risk factors and signs of stroke.[4] However, Kilkenny et al.[4] showed that this knowledge diminished after three months, identifying the

importance of continued exposure to information to improve knowledge retention. Increasing community knowledge of the major risk factors for stroke, such as high blood pressure and diabetes, can result in behavioural change.[4] For example, the Australian ‘Know Your Numbers’ program by Stroke Foundation encouraged people to have their blood pressure checked at various organisations (mainly pharmacies).[5] An evaluation demonstrated that over three months (n=510), 81% of respondents advised to consult their doctor following a blood pressure assessment had sought advice from their doctor, and 44% commenced blood pressure-lowering medication.[5]

Another limitation of the current StrokeSafe presentation model is that it relies on individuals to deliver the education, and therefore is limited in its reach. Our platform will provide our partners at Stroke Foundation with a scalable, cost-effective and easily adaptable platform to significantly augment the reach of their stroke prevention activities.

There are three sequential stages of the Love Your Brain digital platform development and evaluation: Co-design (Stage 1), Implement (Stage 2), and Evaluate (Stage 3). The content and delivery of the Love Your Brain digital platform, which includes a text message system and an online course, was co-designed with stroke knowledge experts and community involvement during 2023 in Stage 1 (Co-design) of Love Your Brain (Monash University Human Research Ethics Committee #35899). In Stage 1, 12 community members and 10 stroke knowledge experts were recruited to complete 8 focus groups (as separate cohorts). The focus groups were conducted to receive ideas and feedback on the structure of, and content for, the digital platform. Elements included “what is stroke?”, signs of stroke, stroke numbers and impact, and risk factor management. Feedback from the focus groups included:

- Use aphasia-friendly language: simple and clear, with no technical terms.
- Use a variety of statistics, personal stories, and expert information to tell a story.
- Videos are preferred over text but make transcripts available.
- Use impact to engage (e.g. “Stroke impacts everything: family, social, and work.”).
- Personalise content as much as possible based on age, sex, location, and risk factors.
- Provide options for simplified or advanced information (e.g. provide trusted weblinks for further information).
- Emphasise the importance of going to the doctor for stroke prevention.
- Acknowledging that enacting change is difficult.

The feasibility and acceptability of Love Your Brain was tested in a pilot trial in Stage 2

(Implement) throughout 2024 (Monash University Human Research Ethics Committee #41176). From April to July 2024, 31 participants were randomised (control: n=11, online course: n=9, text messages: n=11). 30 (97%) completed the intervention (1 withdrew), and 24 (77%) completed the 12-week survey (control: n=8, online course: n=7, text messages: n=9). Eleven participants were recruited directly from StrokeSafe presentations, and 20 via pre-recorded presentations to a wider audience. Feedback data were collected from a participant satisfaction survey (n=24), and interviews/focus groups with participants (control: n=1, online course: n=2, text messages: n=1) and StrokeSafe presenters (n=5).

Participant: overall participants viewed their involvement in the pilot as positive, with most stating they would recommend to others (control 57%, online course 86%, text messages 86%). The different modalities (videos, text, quizzes, images) and “flexibility” of the **online course** were valued. Navigation was described as easy, an many preferred the “short, sharp” videos. Overall, the online course was described as "excellent," "accessible," "relevant," "informative," and "interesting." Suggestions included more depth on cholesterol and progress indicators. **Text messages** were seen as "convenient," with specific, personalised texts most impactful. Timing of the text messages was suitable, and participants appreciated consistency in sender numbers but had concerns about clicking web links. **Control group emails** were easy to access, but could get lost in the volume of emails received. **General challenges noted:** Surveys (baseline, 12-week completion) were lengthy and difficult to complete, especially by phone; reminders to engage with the digital platform may help. Some found the information repetitive and videos occasionally loaded slowly.

StrokeSafe Presenters’ feedback: Drop-in sessions and focus groups provided valuable recommendations, including introducing Love Your Brain earlier, explaining what a digital platform is clearly, increasing the detail of what the platform entails for the participant, and creating a more personal recruitment video. Presenters felt adequately supported through training and feedback, but suggested improving communication about the support available for presenters (handbook, drop-in sessions). Participants highlighted how engagement in StrokeSafe presentations may influence recruitment, with suggestions to the recruitment video and strategies.

Improvements for Stage 3: Based on feedback, enhancements we made include shorter surveys, transitioning to a single-blinded trial (from double-blinded), focusing on recruitment, strengthening follow-up processes, and refining participant communication strategies. This feedback has been instrumental in refining the program and recruitment strategies.

This protocol and application for ethics approval relates specifically to **Stage 3 (Evaluate), a fully powered effectiveness randomised controlled trial.** This trial will evaluate whether

Love Your Brain helps people identify and manage their risk factors, for example: by seeing their general practitioner (GP) for an assessment of risk factors; having increased knowledge of risk factors and signs of stroke; and adopting healthy behaviours for controlling risk factors of stroke. The Stage 3 Protocol includes an outline of the planned methods to assess the effectiveness of the online course and text message aspects of the digital platform, as well as the minimal active control arm. We aim to complete Stage 3 (Evaluate) by December 2025 (see Section 8. Project timeline).

3. AIMS AND OBJECTIVES

The overarching objective of the Love Your Brain trial is to improve the knowledge of risk factors for stroke and the uptake of healthy behaviours needed to prevent stroke in the community. In Stage 3 (Evaluate) the objective is to evaluate the effectiveness of the Love Your Brain digital platform through a randomised controlled trial and process evaluation and economic evaluation carried out in parallel to the main trial. As displayed in Figure 2, Love Your Brain will have three-arms: text message intervention (developed by Monash University), online course intervention (developed by Menzies Institute for Medical Research at the University of Tasmania), and a minimal active control involving five emails (based on emails sent by Stroke Foundation for another initiative). The two intervention arms will each be compared to the minimal active control arm. A direct comparison between the text message intervention and the online course intervention is not intended, as both will be available concurrently in real-world settings.

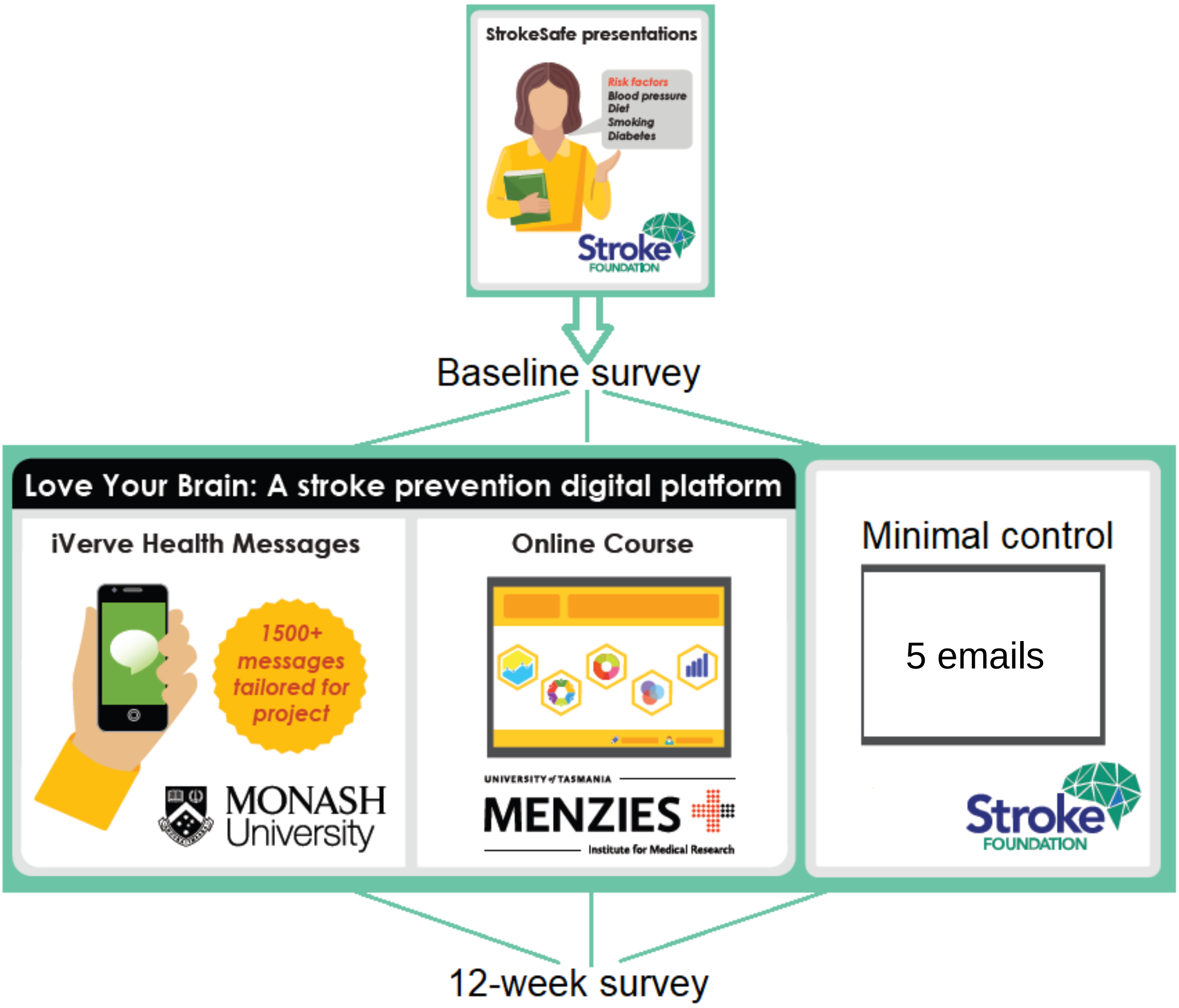

Figure 2: Love Your Brain three-arm randomised controlled trial

The specific aims of this randomised controlled trial are to:

1. Determine whether Love Your Brain improves:
    - visits to a medical practitioner for cardiovascular risk assessment and management from either a GP or specialist, within 3 months of randomisation (Primary Outcome)
    - the knowledge of signs and risk factors for stroke (Secondary Outcome)
    - the maintenance and uptake of healthy or risk-modifying behaviours (Secondary Outcome)
    - adherence to medications (Secondary Outcome)
2. Produce complementary evidence from a process evaluation and cost-effectiveness economic evaluation to support replication in other settings/contexts and for scaling and priority setting decisions.

4. METHODS

We will evaluate the Love Your Brain digital platform in a prospective, parallel three group, single-blinded (statistician and research staff who interact with participants), individual-randomised controlled trial with intention-to-treat analysis. The details of the interventions, trial design, methods and reporting are provided in the following sections. We will also register the trial prospectively on the ANZCTR.

4.1 Participants

Inclusion criteria:

- No history of stroke or other major cardiovascular event (self-reported; includes heart attack/myocardial infarction, coronary artery bypass surgery)
- Aged ≥45 years
- Able to communicate in English
- Residing in Australia
- Able to access the internet and a smartphone
- Have watched a StrokeSafe presentation (in-person or online) in the last three months

4.2 Intervention Arms

All three arms will receive three administrative messages, including a welcome message, 12-week completion survey invitation, and thank you message. All participants will be recontacted once the interventions are publicly available, with an estimated release date in 2026.

4.2.1 Online Course Intervention

The online course is embedded into a Massive Open Online Course (MOOC) infrastructure at the University of Tasmania. The online course contains seven core modules with nine elective modules to be delivered over eight weeks. It follows the process undertaken for previous online courses developed at the University of Tasmania,[6] and is enhanced through the use of simple key messages at the beginning of each module, including videos and personal stories, along with more detailed content.

The online course content and delivery were informed by existing national and international resources and guidelines for preventing stroke (e.g. guidelines produced by Stroke Foundation, World Stroke Organization and the American Heart Association) and co-designed (Stage 1: Co-design). A framework to guide the selection of resources is outlined in **Appendix B**. As detailed in **Appendix C**, the core modules include the definition and

epidemiology of stroke, signs of stroke, a general overview of risk factors for stroke, with elective modules covering specific details about risk factors for stroke relevant to the individual participant, and an action plan. The online course content was checked for a reading level below grade 8 (equivalent to a reading level of adolescents aged 13 to 14 years).

Participants randomised to the online course will be automatically enrolled by the University of Tasmania research team. Email communication via REDCap will include links to access the modules. Once enrolled, online course participants will have access to the content for 8 weeks, within the allocated 12-week intervention period from randomisation. Once enrolled, online course participants will receive regular automated email communication from the University of Tasmania encouraging engagement and completion of the online course.

4.2.2 Text Message Intervention

Text message development was informed by an existing messaging system for people living with stroke,[7] existing national and international resources and guidelines for preventing stroke (e.g. guidelines produced by Stroke Foundation, World Stroke Organization, and the American Heart Association), and co-design (Stage 1: Co-design). A framework to guide the selection of resources is outlined in **Appendix B**. The text messages will be delivered via short messaging service (SMS) by default. Participants can change their preferences to receive text messages by email using the link in the welcome email. Text messages will be two-way, allowing participants to respond and engage with the research team. The number of text messages delivered over the 12 weeks will range between 28 and 58, dependent on the number of risk factors the participant chooses to learn more about in the baseline survey. Participants allocated to the text message arm will be allowed to change the risk factor selection, and therefore the number of messages received, at the time of randomisation. The connection between the number of risk factors and the number of messages will be explained. The personalisation link can be used any time during the 12-week intervention.

As detailed in **Appendix D**, the content aligns with that presented in the online course and includes the definition and epidemiology of stroke, signs of stroke, a general overview of risk factors for stroke, specific details about risk factors for stroke relevant to the individual participant, and an action plan. The text message content was checked for a reading level below grade 8 (equivalent to a reading level of adolescents aged 13 to 14 years).

4.2.3 Minimal Active Control

The minimal active control arm has been designed to give participants a sense of participation in the trial despite being randomised to receive minimal intervention. Minimal participant communication (rather than no communication) may mitigate dropouts by

decreasing dissatisfaction of being assigned to the minimal active control arm. The development of the control arm as a minimal intervention was informed by The Stroke Recovery and Rehabilitation Roundtable's recommendations for control intervention design.[8]

The minimal active control arm will receive five messages via email delivered over the 12-week intervention period, delivered approximately every two weeks. The minimal active control messages are based on emails originally sent by Stroke Foundation for the Victorian Blood Pressure Van Program in conjunction with Ryman Healthcare. These messages contain information and resources available on Stroke Foundation website. As detailed in **Appendix E**, the content includes information on stroke risk factors (blood pressure, diet, alcohol, exercise, smoking) and links to relevant pages on Stroke Foundation website.

4.3 Sample Size, Recruitment, Consent and Randomisation

4.3.1 Sample size

For the randomised controlled trial, we will seek to recruit ~1000 participants (~333 per arm).

We will have adequate power to detect meaningful differences in the primary outcome. Based on the results of Stage 2 (Implement), we anticipate we will recruit one-third of participants from live StrokeSafe presentations, and two-thirds from other means who will watch a pre-recorded StrokeSafe presentation on demand (see 4.3.2 Recruitment). Conservatively, 12,000 people will attend live StrokeSafe presentations in the recruitment year. Based on previous research,[9] 20%-30% will agree to participate with potential sample between n=2,400 and n=3,600. As shown in the table, assuming 80% power with α 0.05, we will have an adequate sample to detect a 30% relative increase in GP health checks in intervention groups with 40% prevalence of the outcome in the control group, this being a conservative estimate from published Australian data.[10,11] Using the United Kingdom modelling, increases of this magnitude in attending health checks are estimated to have meaningful effects on population level health and economic outcomes.[12] Based on Howard et al.[13], we are using a common control group with no adjustment for family-wise type-I error rate because our hypotheses do not inform a common claim of effectiveness for each of the interventions.

While the total sample size of 894 is required (298 per arm), we will aim for 1,000 participants (~333 per arm).

Prevalence GP health assessment in control group	Relative intervention effect	Prevalence GP health assessment intervention group	n/group needed including 10% drop out	Total n
40%	30%	52%	298	894

4.3.2 Recruitment

Recruitment for Love Your Brain has been tested through a sub-study of Stage 1 (Co-design; approved by Monash University Human Research Ethics Committee #35899) and the interim feasibility and acceptability analysis study conducted during Stage 2 (Implement; approved by Monash University Human Research Ethics Committee #41176).

Recruitment for Love Your Brain will occur through two pathways:

1. Live StrokeSafe presentations
2. Pre-recorded StrokeSafe presentations

4.3.2.1 Recruitment through the live StrokeSafe presentations

StrokeSafe presenters will promote enrolment in the Love Your Brain trial within their presentation. A communication strategy with StrokeSafe presenters is outlined in **Appendix F**.

In short, recruitment materials (**Appendix G**) include:

- A video by the Principal Investigator (Prof Monique Kilkenny), and people with lived experience of stroke
- An invitation/recruitment slide
- A hard copy flyer for attendees to take with them.

The slide and flyer will include the Love Your Brain email address and phone number and a link to join the trial through the REDCap. The video and slide will be embedded within the StrokeSafe presentation. If the venue does not have the appropriate technology to play the video, attendees can scan the quick response (QR) code on the recruitment slide or hard copy flyer to watch the video.

A paper sign-up sheet may also be provided at each venue for participants to provide their name, email, and phone number to be contacted by the research team about the trial. Members of the research team may also attend some StrokeSafe presentations to answer questions and promote the trial to attendees.

4.3.2.2 Recruitment through the pre-recorded StrokeSafe presentations

A pre-recorded StrokeSafe presentation will be embedded within the Love Your Brain REDCap for participants to watch on demand, prior to enrolment. Participants can access the Love Your Brain REDCap using the QR code or links provided in all recruitment

materials.

Recruitment material will be distributed through the following channels:

- Email to existing networks
- Email to people who have expressed interest in participating in Love Your Brain after recruitment closed for Stage 2 (collected with approval by Monash University Human Research Ethics Committee #41176)
- Email to the people who have participated in similar trials and provided permission to be contacted for future research
- Flyers to be displayed in public places and at events (e.g., Stroke Foundation events)
- Social media
- Media

An external marketing company will be engaged to develop recruitment materials for Love Your Brain. A sample of indicative recruitment materials are displayed in **Appendix G.** While the key content will not change significantly, the exact recruitment materials are subject to change.

4.3.3 Consent

There will be two separate digital consent forms.

4.3.3.1 Love Your Brain Consent

The first consent form is to participate in the trial and will be administered before commencing the baseline survey. Digital e-consent via REDCap will be used for participation in Love Your Brain. The QR code on all marketing materials will link directly to the Love Your Brain consent form (**Appendix H**), with the explanatory statement embedded (**Appendix I**). Following consent, the participant will continue to the baseline survey.

4.3.3.2 Services Australia Consent

The second consent form is to link survey data with Services Australia data (https://www.servicesaustralia.gov.au/statistical-information-and-data) to allow validation of medical practitioner attendance (primary outcome) using Medicare Benefit Schedule (MBS) and Pharmaceutical Benefits Scheme (PBS) item numbers. Providing consent to link to Services Australia data is separate to providing consent to complete the trial (i.e. consenting to data linkage it is not mandatory).

Preferably, digital e-consent via REDCap will be used for linked data consent. At the end of the baseline survey, participants will be provided information about data linkage with Services Australia, including linkage and provision of MBS and PBS data related to the trial

aims around GP attendance for risk factor assessment and medications related to stroke prevention. Content of the consent form (**Appendix J**) and participant information sheet (**Appendix K**) is specified by Service Australia, and this consent form will be shared with Services Australia to facilitate linkage.

4.3.4 Randomisation

Participants will be randomised into the three intervention arms in 1:1:1 ratio. Randomisation will be undertaken through the REDCap, with stratification balancing by age (45 to >65, ≥65 years) and gender (man, woman, non-binary/gender diverse/prefer not to say). The randomisation table, which includes the allocation sequence, block sizes, and stratification balancing, will be developed by the project statistician (Dr Muideen Olaiya; **Appendix M**).

After randomisation, the same three administrative messages will be sent to participants of all arms at the start and end of the trial. These include a welcome message including a summary of their potential risk factors for stroke (based on their baseline survey responses), a 12-week completion survey invitation, and a thank you message at the end of their participation. Participants randomised to the minimal active control and online course arms will receive the administrative messages by email. Participants randomised to the text message arm will receive the welcome message by email (due to its length), and the 12-week completion survey invitation and thank-you messages via SMS.

4.3.5 Blinding

The trial has a single-blinded trial design, where the statistician and research staff who interact with participants are blinded. The participants and other research staff are unblinded and aware of the three randomisation arms. Therefore, staff and participants are unblinded to group allocation. Participants who indicate they are interested in participating in future research or receiving the results of the trial, or contact us to indicate they are interested in accessing the other intervention arms will be contacted at the end of the trial in 2026. They will be offered the opportunity to access the intervention arms once they become publicly available. The trial is described in the patient information and consent form in general terms as a “digital platform”. Specifically, intervention approaches are broadly defined in the participant consent form as “The trial provides evidence-based information delivered over 12-weeks either by email or text messages or an online course. The information can be accessed on your smart phone, laptop/computer, or tablet.” With the additional detail in the Explanatory Statement “You will not be able to choose how you receive the information as it is chosen at random.” The data analyst (statistician) and research staff who interact with participants will be blinded to ensure analyses and participant interactions are not influenced during or after the trial, for example, by conscious or unconscious selection of statistical tests

and reporting.

4.3.6 Withdrawal

Participants will be provided with the opportunity to cease participation at any time by responding “STOP” to any email or SMS, or contacting the Project Coordinator. When a “STOP” message or other contact is received, a member of the research team will contact the participants to discuss whether they prefer to withdraw from the trial, or to modify their preferences for the intervention (e.g., receive fewer text messages per week, change their elected online course modules).

To withdraw from the trial, a verbal indication is sufficient. Participants will be asked to complete a withdrawal form (which can be emailed to the participant or completed by a member of the research team on their behalf; **Appendix N**) via REDCap to ascertain any adverse events, and also provide an opportunity to describe their reason for withdrawal (voluntary), and provide feedback or improvements for the Love Your Brain digital platform (voluntary).

To withdraw consent for data linkage, participants will be asked to complete a Services Australia withdrawal of consent form (**Appendix O)**.

4.3.7 Follow-up procedures

The Project Coordinator will be the primary point of contact for participants, and the primary administrator of the Love Your Brain email account (LoveYourBrain@monash.edu) and phone number. In general contact will follow the Dillman protocol[16], initiated with emails (days seven and 14), then phone calls (days 15 to 21 or 28) with SMS potentially used to notify individuals of an upcoming phone call.

4.3.7.1 Expressions of Interest

If people contact the research team by email or phone to express interest in participating, the Project Coordinator will send a personalised email (based on **Appendix L**) which links them to the online consent form, explanatory statement and baseline survey. If the person then does not commence the baseline survey, a member of the research team will send two reminder emails (days seven and 14). On day 15 to 21, a researcher will phone participants to answer any unanswered questions they may have, assess their interest in continuing with participation, and offer to assist by completing the survey with them (**Appendix Q**). If the participant cannot be reached, daily weekday phone calls may be attempted between days 15 and 28.

4.3.7.2 Prior to Randomisation

For participants who give consent and provide their contact details, but do not complete the

baseline survey, automated reminders will be sent through REDCap by email on days seven and 14. On day 15 to 21, a researcher will phone participants to answer any unanswered questions they may have, assess their interest in continuing with participation, and offer to assist by completing the survey with them (**Appendix Q**). If the participant cannot be reached, daily weekday phone calls may be attempted between days 15 and 28. The baseline survey will not be accessible after 35 days from commencing the survey.

4.3.7.2 After Randomisation into the Online Course

Participants randomised to the online course will be automatically enrolled and receive a “welcome email” from the University of Tasmania team. Throughout the 8-week access period for the online course, participants will receive email reminders to return to the online course to complete or revisit modules (weeks 2, 4, 6, and 7). By day 15, two emails will have been sent to participants who have not completed the first module. On day 15 to 21, a researcher will SMS and phone participants to assess their interest in continuing with participation, and troubleshoot any technical issues that might be preventing them accessing the online course.

4.3.7.3 At 12-week survey

For participants who have not started the 12-week survey (or who have started but not completed the survey), two automated reminders will be sent through REDCap by email (minimal active control and online course arms) or SMS/email (text message arm, depending on preference) on days seven and 14 after trial completion. On day 15 to 21, a researcher will phone participants to answer any unanswered questions they may have, assess their interest in continuing with participation, and offer to assist by completing the survey with them (**Appendix Q**). If the participant cannot be reached, daily weekday phone calls may be attempted between days 15 and 28. The 12-week survey will not be accessible after 35 days from the day they are invited to complete the 12-week survey.

5. DATA COLLECTION AND PROCESS EVALUATION

Methods and results will be reported in compliance with the CONSORT 2010 Statement.

5.1 Baseline Survey

Consent will be embedded in the digital baseline survey (**Appendix H**). Following consent, the baseline survey (**Appendix P**) will include:

Survey	Justification	Questions*	Estimated duration

Eligibility	To determine if the participant meets the trial eligibility criteria	8	1 minute
StrokeSafe Presentation	To identify recruitment source and receive feedback on the presentation<br>OR<br>Watch a pre-recorded StrokeSafe presentation	4<br><br>0	1 minute<br><br>10 minutes
Participant information, including contact preferences	To administer the digital platform	6	1 minute
Demographic information	To ensure digital platform is relevant to all sub-groups, identify confounding factors	7	2 minutes
Medical practitioner attendance for cardiovascular risk factor assessment	Primary outcome	2 (+3)	2 minutes
Stroke Knowledge Test[14]	Secondary outcome	20	5 minutes
Identification of health and lifestyle risk factors	Secondary outcome	20 (+15)	6 minutes
Quality of life (EQ-5D-5L-Psychosocial)[15,16]	Secondary outcome, economic evaluation	10	2 minutes
Selecting healthy choices to manage risk factors	To administer the digital platform	1	<1 minute
Health behaviours	Secondary outcome, economic evaluation	3	<1 minute
Medication Adherence Rating Scale (MARS-5)[21]	Secondary outcome, economic evaluation	1 (+6)	2 minutes
Healthcare resource utilisation	Economic evaluation	10 (+3)	2 minutes

*Parenthesised numbers indicate additional questions that may be presented to participants based on branching logic to previous questions in the survey

5.2 12-Week Completion Survey

The 12-week completion survey will repeat the baseline surveys (**Appendix P**):

- Medical practitioner attendance for cardiovascular risk factor assessment
- Stroke Knowledge Test[18]
- Identification of health and lifestyle risk factors
- Quality of life (EQ-5D-5L-Psychosocial)[19,20]
- Health behaviours
- Medication Adherence Rating Scale (MARS-5)[21]
- Healthcare resource utilisation

In addition, we will include:

- Adverse events (if the participant self-reports presentation to emergency department or admission to hospital; **Appendix R**), and
- Satisfaction and evaluation survey (overall satisfaction, willingness for further contact regarding experience; **Appendix S**).

5.3 Data Linkage

Survey data will be linked to Services Australia data (MBS and PBS) to validate the primary outcome, validate medication adherence (secondary outcome), and to provide information on resource use and out of pocket costs for the economic evaluation. Linkage will only occur for participants who provide consent (**Appendix J**).

5.4 Program Evaluation

A mixed method process evaluation will be conducted in parallel to the effectiveness randomised controlled trial to describe the delivery of the interventions, and obtain feedback to understand the acceptability and feasibility of the intervention, and potentially how and why the intervention may be effective (causal pathways). Potential barriers and facilitations to implementation of the digital platform, and suggestions for improvement will also be sought. The process evaluation will be guided by the Practical, Robust Implementation and Sustainability Model (PRISM) framework{Glasgow RE, 2024 #40} which considers the external environment, organisational context, intervention characteristics and implementation and sustainability infrastructure that may influence the trials success in real-world settings. There are two components to the process evaluation. A formative assessment undertaken at the start of the trial and the final summative evaluation at the conclusion of the trial.

5.4.1 Formative Assessment

After the first 80-100 participants have completed their 12-week surveys, a researcher independent of the trial delivery will conduct a formative assessment to review study procedures and methods (recruitment, consent, randomisation, survey completion), and the feasibility and fidelity to improve the quality and efficiency of the trial (including analysis of participant surveys; interviews/focus groups with participants, StrokeSafe presenters, and study team; and process metrics). Subsequent protocol amendments will occur as necessary (including ethics review as required). The formative assessment will include relevant study documentation, analysis of participants' perceptions collected to date from the 12-weeek satisfaction and evaluation survey (**Appendix S**). A focus group with select study researchers/team involved with recruitment and participant focus group/interviews would be optional should it be deemed beneficial based on feedback from the satisfaction and evaluation surveys.

Data for the process evaluation will be collected from a range of sources including study documentation, user metrics related to the online course and access to weblinks, completion of surveys, in addition to a participant satisfaction and evaluation survey (**Appendix S)** and focus group/interviews (**Appendix T**).

5.4.2 Study documentation and metrics

Study documentation and metrics will be used to particularly assess feasibility and fidelity. Specific data will include:

- Number of participants recruited
- Proportion allocated to each arm: disadvantaged areas, rural areas, culturally and linguistically diverse communities
- Completeness of surveys (baseline and 12-week completion)
- Time taken to complete surveys
- Retention of participants in the 12-week trial
- Amount (dose) of the interventions delivered (measured by the number of text messages received per week, or time spend engaging with the online course, or time spent self-reported in the 12-week survey)
- Number, reasons and modality of participant communication
- Number and reasons for respondents commencing and withdrawing
- Number of SMS messages that failed to send
- Number of participants who started, completed or failed to complete the online

course

- Relevant trial documents (e.g. meeting minutes, communications, research notes).
- Metrics pertaining to the intervention arm engagement (e.g. link clicks, online course quiz completions).
- Number of StrokeSafe presentations attended by the research team
- Costs of delivering the intervention (e.g. online course infrastructure, SMS credits)

In addition, we will utilise Stroke Foundation data to report on the:

- Total number of attendees at StrokeSafe presentations during the trial
- Proportion of attendees recruited
- StrokeSafe presenter socio-demographics (age, sex, location) and connection to stroke (e.g. person with lived experience, carer, health professional).

To assess the feasibility and acceptability of the approach used in the minimal active control arm we will monitor the rates of drop-outs and compare to the rates in intervention arms.

5.4.3 Satisfaction and Evaluation Survey

All participants will be invited to complete an electronic satisfaction and evaluation survey at the end of their 12-week intervention. (**Appendix S**; follow-up procedure outlined in section 4.3.7.3). Survey items are specific to components of the intervention in addition to drawing on established models/theories to specifically explore factors influencing use of technology.{Venkatesh V, 2003 #41} The survey includes both closed and free text responses to ascertain participant perceptions of the acceptability, and satisfaction with the digital program and any potential challenges and areas for improvement.

5.4.4 Focus Groups

Purposefully sampled participants from each arm will be invited to attend an online focus group (or 1:1 interview if preferred) specific to their arm (n=3) after their completion of the trial (**Appendix T**). Sampling will be based on their indication of interest in participating (from the satisfaction and evaluation survey) and survey responses. In addition, we may invite StrokeSafe presenters (or others involved in recruitment) to attend a focus group (n=1; **Appendix T**).

Focus groups will evaluate perceptions and experiences of the feasibility, acceptability, and fidelity of the digital program and trial process. Outcomes will provide an understanding of how and why the intervention arms may be effective, identify potential barriers and facilitators to implementation, and offer suggestions (if any) for the remainder of the trial. Focus group

interviews will be conducted by a researcher independent of study procedures. The interviews will take about 60 minutes to complete and be conducted via online video conferencing or telephone. These interviews will be recorded and transcribed verbatim, with notes taken to record other details not evident on the recording. See **Appendix T** for extended information.

Ultimately, the findings from this process evaluation will be used to inform future co-design work, and will assist in unpacking reasons for outcome findings, including potential barriers, facilitators and contextual influences, which are important to support future translation.

5.4.3 Economic Evaluation

To determine the potential cost-effectiveness of Love Your Brain we will summarise the program delivery costs and the costs to participants. The costs of providing the project and intervention arms will be based on information collected for the operational costs associated with the delivery of the interventions as tracked in relevant finance reports, and will include the costs of consumables and presenters, as well as the relevant Stroke Foundation staff. Unit prices for resources used by participants will be obtained from the most relevant and contemporary Australian sources. The findings from this evaluation will be used to develop future business case for establishing and maintaining Stroke Foundation's "Love Your Brain: A stroke prevention digital platform".

6. DATA ANALYSIS

The statistical analysis plan is provided as **Appendix U,** which will likely be submitted for publication. In brief, the statistician will be blind to group allocation and the primary outcome is attendance at a medical practitioner for cardiovascular risk assessment and management. The primary analysis will use intention to treat analysis[23] and a secondary per-protocol analysis will also be performed. The two intervention arms will each be compared to the one minimal active control arm. A direct comparison between the text message intervention and the online course intervention is not intended, as both will be available concurrently in real-world settings. The primary outcome is attendance at a medical practitioner for cardiovascular risk assessment and management from either a GP or specialist within 3 months of randomisation for each intervention arm.

A mixed method process evaluation will be conducted using following analyses as appropriate for the type of data.

Quantitative analyses for survey responses and user metrics will be undertaken using a current version of STATA for Windows (current version) and Microsoft Excel (current version). Descriptive statistics (e.g., frequencies, means) will be used as appropriate for the

type of data.

Qualitative analysis of free text responses and focus group/interview data will be analysed using thematic and/or content analysis methods.[31] Transcribed data will be thematically coded, using both indictive and deductive approaches as appropriate. A coding tree outlining the themes and sub-themes will be developed as per established methods,[25] and qualitative analysis software (e.g. NVivo) will be used to manage the data. We anticipate double coding up to 10% of transcripts by two independent researchers. De-identified, illustrative quotes may be provided in the reporting of these data.

The findings from each data collection method within the process evaluation will be triangulated as part of the interpretative process to enhance the trustworthiness and credibility of our research findings. Triangulation is the combination of at least two or more theoretical perspectives, methodological approaches, data sources, investigators or data analysis methods.[26] Triangulation strengthens the resultant findings, and will enable a more comprehensive understanding of the impacts of Love Your Brain.

7. ETHICAL CONSIDERATIONS

This trial will be carried out according to the Declaration of Helsinki, the NHMRC National Statement on Ethical Conduct in Research Involving Humans (2007) and the Notes for Guidance on Good Clinical Practice as adopted by the Australian Therapeutic Goods Administration (2000) (CPMP/ICH/135/95) and the International Conference on Harmonisation Good Clinical Practice Guidelines. Ethical approval from Monash University Human Research Committee will be obtained before approaching participants. It is the responsibility of the Principal Investigator to report trial progress to the Ethics Committee as required or at intervals not greater than one year.

Participants can contact the research team throughout the trial. The Project Coordinator will be the primary point of contact for participants and can be contacted via the dedicated Love Your Brain email account (LoveYourBrain@monash.edu) or through the phone number listed on recruitment materials.

7.1 Privacy and confidentiality

Participation is voluntary. All data will be reported in a de-identifiable and aggregated format so that individuals will not be able to be identified. Any quotes reported from qualitative data (e.g. focus groups, email or SMS responses) will not identify individual persons.

7.2 Participant Safety

There are minimal risks for participants of this trial. The main burden on participants is the time required to complete surveys, undertake the online course (approximately one hour

each week) or read text messages (less than two minutes each, up to five messages per week if a participant self-selects that quantity) or read emails (less than ten minutes each, one delivered approximately every two weeks) and initiate action to reduce their risk of stroke.

If a participant requires additional support, upon contact with the research team the participant will be directed to Stroke Foundation's StrokeLine (1800 787 653) as appropriate.

7.2.1 Adverse Events

Serious adverse events (SAEs) are defined according to the Therapeutic Goods Administration *Note for Guidance on Clinical Safety Data Management: Definitions and Standards for Expedited Reporting* (CPMP/ICH/377/95 p.5).

A serious adverse event is any untoward medical occurrence including adverse events listed that:

- Result in death
- Is life-threatening. *Note: The term 'life-threatening' in the definition of 'serious' refers to an event in which the participant was at risk of death at the time of the event. It does not refer to an event, which hypothetically might have caused death if it were more severe.*
- Requires hospitalisation or prolongation of an existing hospitalisation. *Note: In general, hospitalisation signifies that the participant has been detained (usually involving at least an overnight stay) at the hospital or emergency ward for observation and/or treatment that would not have been appropriate in the physician's office or out-patient setting. If a complication prolongs hospitalisation or fulfils any other serious criteria, the event is serious and another event is completed.*
- Results in disability/incapacity. *Note: The term disability means a substantial disruption of a person's ability to conduct normal life functions.*
- Other important medical event. *Note: Medical and scientific judgement should be exercised in deciding whether reporting is appropriate in other situations, such as important medical events that may not be immediately life-threatening or result in death or hospitalisation, but may jeopardise the participant or may require medical or surgical intervention to prevent one of the other outcomes listed in the above definition. These should also be considered serious. Examples of such events are invasive or malignant cancers, intensive treatment in an emergency room or at home for allergic bronchospasm, blood dyscrasias or convulsions that do not result in hospitalisation, or development of drug dependency or abuse.*

Adverse events are considered events of interest for this trial and include any new report of the following that do not meet the criteria for a serious adverse event:

- Any emergency department presentation

All adverse events should be documented in the participant's record in REDCap. Participants will self-report adverse events that have occurred since the time of randomisation at the 12-week completion survey or at the time of withdrawal (**Appendix R**). The participant will provide the diagnosis, start and stop dates, and description of event for each adverse event. A blinded member of the research team will assess the severity, and relationship to the trial in the REDCap. If required, supplemental medical record information provided in the form of de-identified support documentation will be obtained with the participant's consent.

If the event is considered related to the trial intervention, the event will be additionally independently adjudicated a medical monitor independent of the trial. Serious adverse events not related to the intervention will be reported annually to the ethics committee in a summary table. As this is a low-risk trial which does not involve a therapeutic good, and there are no perceived risks to participants, a data safety monitoring committee is not required.

7.2.2 Protocol Breaches

Non-serious breaches (e.g. missed or incomplete trial procedures, trial procedures completed outside protocol timeline) will be reported to the Project Manager and Principal Investigator.
Serious Breaches (e.g. failure to obtain informed consent, enrolment of participants who are ineligible) will be reported to the Monash University HREC using the Protocol Deviation/Violation Report within 7 calendar days of confirmation.

7.2.3 Disclaimer

Love Your Brain is not a therapeutic good and the information in the digital platform does not constitute medical advice. For transparency with participants, the following disclaimer will be added to the explanatory statement and welcome email:
While we make every effort to make sure the information in this digital platform is accurate and informative, the information does not take the place of professional or medical advice. You should obtain advice relevant to your particular circumstances from a health professional. We do not accept any liability for any injury, loss or damage caused by use of the information provided in our digital platform. The information provided, including links to other websites, may include the views or recommendations of third parties and does not

necessarily reflect the views of Stroke Foundation, Monash University or University of Tasmania or indicate a commitment to a particular course of action. You need to make your own decisions about the accuracy, currency, and reliability of information in linked websites.

7.3 Data Security and Storage

Encrypted de-identified data will be shared between trial investigators from Monash University and the University of Tasmania for data analyses, as per the agreed terms in the Collaborative Research Agreement. As required, data will be shared securely between trial investigators via the Monash Secure eResearch Platform (SeRP). To facilitate delivery of the text messages via SMS, the participant's name and phone number will be shared with a third-party text message gateway who host data on Australian servers.

In accordance with the Good Clinical Practice guidelines, identifiable information about the participants will be stored in a separate project file with restricted access. The confidential file will be separate to where the de-identified data are being stored (e.g., participant name and contact information will be separate to program engagement metrics). All data will be stored according to Monash University policy that complies with NHMRC guidelines for the conduct of research.

7.4 Proposed Dissemination of Results

Summative reports will be presented to the Love Your Brain Project Management Committee. A lay summary report will also be available for the participants upon request. The results will also be reported in peer-reviewed publications and presented at scientific conferences.

8. PROJECT TIMELINE

Stage 3 (Evaluate) of Love Your Brain is anticipated to commence in January 2025 and be completed by December 2025, following the suggested timeline below. Provision has been made to extend recruitment (graded cells) if required.

**Stage 3: Evaluate**													
	2024	2025											
	Dec	Jan	Feb	Mar	Apr	May	Jun	Jul	Aug	Sep	Oct	Nov	Dec
Ethics approval													
Recruitment, baseline survey, and randomisation (aim n=1,000)													
12-week completion survey													
Formative analysis													
Randomised controlled trial data analysis													
Process evaluation													
Economic evaluation													

9. REFERENCES

1. Feigin VL, Nguyen G, Cercy K, Johnson CO, Alam T, Parmar PG, Abajobir AA, Abate KH, Abd-Allah F, Abejie AN, et al. Global, Regional, and Country-Specific Lifetime Risks of Stroke, 1990 and 2016. *N Engl J Med*. 2018;379:2429-2437. doi: 10.1056/NEJMoa1804492
2. O'Donnell MJ, Chin SL, Rangarajan S, Xavier D, Liu L, Zhang H, Rao-Melacini P, Zhang X, Pais P, Agapay S, et al. Global and regional effects of potentially modifiable risk factors associated with acute stroke in 32 countries (INTERSTROKE): a case-control study. *Lancet*. 2016;388:761-775. doi: 10.1016/S0140-6736(16)30506-2
3. Kim J., Neville E., Dalli L., Zomer E., Birhanu M., Purvis T., Olaiya MT., Talic S., Kilkenny MF., Cadilhac DA., et al. Economic Impact of Stroke 2024. Melbourne, Australia: 2024.
4. Kilkenny MF, Purvis T, Werner M, Reyneke M, Czerenkowski J, Cadilhac DA. Improving stroke knowledge through a 'volunteer-led' community education program in Australia. *Prev Med*. 2016;86:1-5. doi: 10.1016/j.ypmed.2016.01.015
5. Cadilhac DA, Kilkenny MF, Johnson R, Wilkinson B, Amatya B, Lalor E. The Know Your Numbers (KYN) Program 2008 to 2010: Impact on Knowledge and Health Promotion Behavior among Participants. *Int J Stroke*. 2015;10:110-116. doi: 10.1111/ijs.12018
6. Claflin SB, Gates R, Maher M, Taylor BV. Building a successful massive open online course about multiple sclerosis: A process description. *J Med Internet Res*. 2020;22:e16687-e16687. doi: 10.2196/16687
7. Cadilhac DA, Busingye D, Li JC, Andrew NE, Kilkenny MF, Thrift AG, Thijs V, Hackett ML, Kneebone I, Lannin NA, et al. Development of an electronic health message system to support recovery after stroke: Inspiring Virtual Enabled Resources following Vascular Events (iVERVE). *Patient Prefer Adherence*. 2018;12:1213-1224. doi: 10.2147/ppa.S154581
8. Hayward KS, Dalton EJ, Barth J, Brady M, Cherney LR, Churilov L, Clarkson AN, Dawson J, Dukelow SP, Feys P, et al. Control intervention design for preclinical and clinical trials: Consensus-based core recommendations from the third Stroke Recovery and Rehabilitation Roundtable. *Neurorehabilitation and neural repair*. 2023:15459683231209162-15459683231209162. doi: 10.1177/15459683231209162
9. Redfern J, Thiagalingam A, Jan S, Whittaker R, Hackett ML, Mooney J, De Keizer L, Hillis GS, Chow CK. Development of a set of mobile phone text messages designed for prevention of recurrent cardiovascular events. *Eur J Prev Cardiol*. 2014;21:492-499. doi: 10.1177/2047487312449416

10. Kilkenny MF, Purvis T, Werner M, Reyneke M, Czerenkowski J, Cadilhac DA, National Stroke F. Improving stroke knowledge through a 'volunteer-led' community education program in Australia. *Prev Med*. 2016;86:1-5. doi: 10.1016/j.ypmed.2016.01.015
11. Gonzalez-Chica DA, Bowden J, Miller C, Longo M, Nelson M, Reid C, Stocks N. Patient-reported GP health assessments rather than individual cardiovascular risk burden are associated with the engagement in lifestyle changes: population-based survey in South Australia. *BMC Fam Pract*. 2019;20:173. doi: 10.1186/s12875-019-1066-9
12. Turner LR, Cicuttini F, Pearce C, Mazza D. Cardiovascular disease screening in general practice: General practitioner recording of common risk factors. *Prev Med*. 2017;99:282-285. doi: 10.1016/j.ypmed.2017.03.004
13. Mytton OT, Jackson C, Steinacher A, Goodman A, Langenberg C, Griffin S, Wareham N, Woodcock J. The current and potential health benefits of the National Health Service Health Check cardiovascular disease prevention programme in England: A microsimulation study. *PLoS Med*. 2018;15:e1002517. doi: 10.1371/journal.pmed.1002517
14. Howard DR, Brown JM, Todd S, Gregory WM. Recommendations on multiple testing adjustment in multi-arm trials with a shared control group. *Stat Methods Med Res*. 2018;27:1513-1530. doi: 10.1177/0962280216664759
15. Nunan D HC. Lack of blinding. Catalogue Of Bias. www.catalogueofbiases.org/biases/lackofblinding. 2018. Accessed 10 November 2023.
16. Schulz KF, Altman DG, Moher D. CONSORT 2010 Statement: updated guidelines for reporting parallel group randomised trials. *BMC Medicine*. 2010;8:18. doi: 10.1186/1741-7015-8-18
17. Carter N, Bryant-Lukosius D, DiCenso A, Blythe J, Neville A. The Use of Triangulation in Qualitative Research. . *Oncology nursing forum*. 2014;41:545. doi: 10.1188/14.ONF.545-547
18. Sullivan K, Dunton NJ. Development and Validation of the Stroke Knowledge Test. *Top Stroke Rehabil*. 2004;11:19-28. doi: 10.1310/RED5-V47T-8MJN-JY9H
19. Herdman M, Gudex C, Lloyd A, Janssen M, Kind P, Parkin D, Bonsel G, X. B. Development and preliminary testing of the new five-level version of EQ-5D (EQ-5D-5L). *Qual Life Res*. 2011;20:1727-1736. doi: 10.1007/s11136-011-9903-x.
20. Campbell JA, Ahmad H, Chen G, van der Mei I, Taylor BV, Claflin S, Henson GJ, Simpson-Yap S, Laslett LL, Hawkes K, et al. Validation of the EQ-5D-5L and psychosocial bolt-ons in a large cohort

of people living with multiple sclerosis in Australia. *Quality of Life Research*. 2023;32:553-568. doi: 10.1007/s11136-022-03214-y

21. Chan AHY, Horne R, Hankins M, Chisari C. The Medication Adherence Report Scale: A measurement tool for eliciting patients' reports of nonadherence. *Br J Clin Pharmacol*. 2020;86:1281-1288. doi: 10.1111/bcp.14193
22. Glasgow RE, McKay HG, Piette JD, Reynolds KD. The RE-AIM framework for evaluating interventions: what can it tell us about approaches to chronic illness management? *Patient Educ Couns*. 2001;44:119-127. doi: 10.1016/s0738-3991(00)00186-5
23. Detry MA, Lewis RJ. The Intention-to-Treat Principle How to Assess the True Effect of Choosing a Medical Treatment. *Jama-Journal of the American Medical Association*. 2014;312:85-86.
24. EuroQol G. EuroQol--a new facility for the measurement of health-related quality of life. *Health Policy*. 1990;16:199-208.
25. Mays N, Pope C. Rigour and qualitative research. *BMJ*. 1995;311:109-112. doi: 10.1136/bmj.311.6997.109
26. Thurmond VA. The point of triangulation. *Journal of Nursing Scholarship*. 2001;33:253-258. doi: DOI 10.1111/j.1547-5069.2001.00253.x
27. O'Donnell MJD, Chin SL, Rangarajan SM, Xavier DP, Liu LP, Zhang HP, Rao-Melacini PM, Zhang XM, Pais PP, Agapay SB, et al. Global and regional effects of potentially modifiable risk factors associated with acute stroke in 32 countries (INTERSTROKE): a case-control study. *Lancet*. 2016;388:761-775. doi: 10.1016/S0140-6736(16)30506-2
28. Malterud K, Siersma VD, Guassora AD. Sample Size in Qualitative Interview Studies: Guided by Information Power. *Qual Health Res*. 2016;26:1753-1760. doi: 10.1177/1049732315617444
29. Moore GF, Audrey S, Barker M, Bond L, Bonell C, Hardeman W, Moore L, O’Cathain A, Tinati T, Wight D, et al. Process evaluation of complex interventions: Medical Research Council guidance. *BMJ*. 2015;350:h1258-h1258. doi: 10.1136/bmj.h1258
30. National Health and Medical Research Council, Australian Research Council and Universities Australia. Payment of participants in research: information for researchers, HRECs and other ethics review bodies. . Commonwealth of Australia. www.nhmrc.gov.au/about-us/publications/payment-participants-research-information-researchers-hrecs-and-other-ethics-review-bodies. 2019. Accessed Sep 26.
31. Mays N, Pope C. Qualitative Research in Health Care: Assessing Quality in Qualitative Research. *BMJ*. 2000;320:50-52. doi: 10.1136/bmj.320.7226.50
